# Aconite as a Local Anæsthetic

**Published:** 1875-05

**Authors:** Paul W. Allen


### ARTICLE X.

### *Aconite as a Local Anæsthetic.*

Dental Caries.—In these cases the tincture is probably efficacious as an anæsthetic just in proportion as it reaches the nerve itself. Most dentists and physicians have often applied it in toothache, and it has given temporary relief in those cases where the nerve has been exposed. A few applications will sometimes destroy the vitality of the nerve entirely.

To Fill Teeth Without Pain.—This is the use of aconite to which I now wish to direct the attention of physicians and dentists. To me it is a new idea. Is it practicable? Years since, I was familiar with current dental literature, but not now; and perhaps I am suggesting something that may be familiar to others, and which may form an item of instruction in Dental Colleges. I have no doubt that the tincture of aconite root would temporarily so benumb sensitive dentine, that it might be excavated without pain, but this I do not know from actual experience. But that it will benumb an exposed nerve while we apply escharotic agents, I seem to have determined in a recent case. In my earlier life as a country physician, I devoted a portion of my time to dental operations, and so a very few family friends still insist that I shall "fill this tooth." And a few days since, in excavating a cavity on the articulating surface of a small molar, my drill reached the pulp. The opening was quite small, and at the side, rather than upon the end of the nerve cavity. The pulp bled freely and I lightly filled the excavation with cotton. There was but little pain for two days, but the cotton could not be even slightly pressed upon without the darting stab of pain. The cotton was then removed with the intention of destroying the nerve with acetate of morphia and arsenic; but this is commonly so painful that it occurred to me to first *anæsthetize the nerve with aconite.* Cotton, saturated with tincture, was placed in the excavation. It produced only slight and momentary pain, and in fifteen minutes it was removed. Another pledget, wet in the tincture and well covered with the morphine and arsenic, was now introduced. It remained in the cavity three hours, but produced no pain. It was then taken out, and as all discolored dentine had been previously removed, I decided to fill with gold, at once. It was plugged, the pressure upon the pellets not being made at first directly in the line towards the opening to the pulp; but afterward placed as solidly in every direction as serrate pointed instruments could be made to pack it. There has since been no pain or other evidence of inflammation. This is only one case, but it seems entirely successful. The structure near the nerve was hard and clear. I did not excavate the nerve-cavity, but the patient informs me that he repeatedly inserted a pin into it, without pain; and the nerve was therefore evidently destroyed. It is unquestionable that intense pain would have been produced by the pin, as well as by the escharotic, or along with it, or both.

May we not thus destroy any dental nerve without pain? If so, this suggestion may be instructive to physicians, may be of great value to the dentist, and may save immense suffering to those who have such teeth to be filled.

This suggests that other surgical operations, occupying only a small extent of tissue, may, perhaps, be rendered painless by a similar application. If so, aconite may be an agent of much value in developing painless surgery, both in the use of the knife upon limited portions of superficial tissues, and in the use of escharotics to destroy morbid growths.—*Prof. Paul W. Allen, in Medical Eclectic.*

